# Endemic impact of human T cell leukemia virus type 1 screening in bone allografts

**DOI:** 10.1007/s10561-016-9586-1

**Published:** 2016-09-27

**Authors:** Yasuhiro Ishidou, Kanehiro Matsuyama, Eiji Matsuura, Takao Setoguchi, Satoshi Nagano, Hironori Kakoi, Masataka Hirotsu, Ichiro Kawamura, Takuya Yamamoto, Setsuro Komiya

**Affiliations:** 1 Departments of Medical Joint Materials, Graduate School of Medical and Dental Sciences, Kagoshima University, 8-35-1 Sakuragaoka, Kagoshima, 890-8520 Japan; 2 Departments of Neurology and Geriatrics, Graduate School of Medical and Dental Sciences, Kagoshima University, Kagoshima, Japan; 3 The Near-Future Locomotor Organ Medicine Creation Course, Graduate School of Medical and Dental Sciences, Kagoshima University, Kagoshima, Japan; 4 Departments of Orthopedic Surgery, Graduate School of Medical and Dental Sciences, Kagoshima University, Kagoshima, Japan

**Keywords:** Human T-lymphotropic virus type 1, Allograft, Bone bank, Disease transmission

## Abstract

Allograft bone is a widely used as a convenient tool for reconstructing massive bone defects in orthopedic surgery. However, allografts are associated with the risk of viral disease transmission. One of the viruses transmitted in this manner is human T-lymphotropic virus type 1 (HTLV-1), which is found worldwide but is unevenly distributed. The southwestern parts of Japan are a highly endemic for HTLV-1. We investigated the HTLV-1 seroprevalence in candidate allograft donors at the regional bone bank in Kagoshima, Japan during its first 5 years of service. Between 2008 and 2012, we collected 282 femoral heads at the Kagoshima regional bone bank from living donors with osteoarthritis of the hip joint. Among the 282 candidate donors, 32 donors (11.3 %) were seropositive for anti-HTLV-1 antibody; notably, this prevalence is higher than that reported for blood donors in this area. Additionally, to determine if HTLV-1 genes are detectable after processing, we examined the bone marrow of the femoral heads from seropositive donors by conducting PCR assays. Our results confirm the existence of viral genes following the heat treatment processing of the femoral heads. Therefore, it is important to inactivate a virus completely by heat-treatment. Together, our findings highlight the importance of HTLV-1 screening at bone banks, particularly in HTLV-1-endemic areas such as southwest Japan.

## Introduction

Allograft bone is a widely used as a convenient tool for reconstructing massive bone defects in orthopedic surgery (Engh and Ammeen 2007; Komiya et al. 2003; Rogers et al. 2012; Urabe et al. 2007). However, allografts are associated with the risk of viral disease transmission, including the transmission of human immunodeficiency virus type 1 (Li et al. 2001; Simonds et al. 1992), hepatitis C virus (HCV) (Conrad et al. 1995), and human T-lymphotropic virus type 1 (HTLV-1) (Sanzén and Carlsson 1997).

HTLV-1 is a retrovirus associated with adult T-cell leukemia/lymphoma (ATL) and HTLV-1-associated myelopathy/tropical spastic paraparesis (Edlich et al. 2003; Izumo et al. 2000). Although this virus is distributed globally, its distribution is uneven. Highly endemic regions include the southwestern parts of Japan, sub-Saharan Africa, South America, the Caribbean, and foci in the Middle East and Australo-Melanesia. The main causes of HTLV-1 transmission include blood transfusion, breastfeeding, and sexual contact (Gessain and Cassar 2012; Manns et al. 1999).

Kagoshima Prefecture is the most southwestern prefecture of Japan, and based on data from blood donors, it has a high seroprevalence of HTLV-1 (Vrielink and Reesink 2004). However, the HTLV-1 seroprevalence of allograft donors in this area has not been reported. The femoral heads procured for use in allografts were disinfectioned by heat treatment and were cryopreserved until transplant. Prior to this study, it was unknown if these processing procedures eliminated the viral genes in femoral heads that were procured from a virus carrier.

Here, we investigated the seroprevalence of HTLV-1 in donor candidates at Kagoshima’s regional bone bank during its first 5 years of service. Additionally, we used PCR to determine whether or not the HTLV-1 gene exists in the bone marrow of femoral heads from seropositive donors after the donated bones have been processed. The aim of our research was to clarify the seroprevalence of HTLV-1 in living donor candidates within an endemic area and to confirm the importance of thermal disinfection for preventing any transmission of HTLV-1.

## Materials and methods

**Fig. 1:**
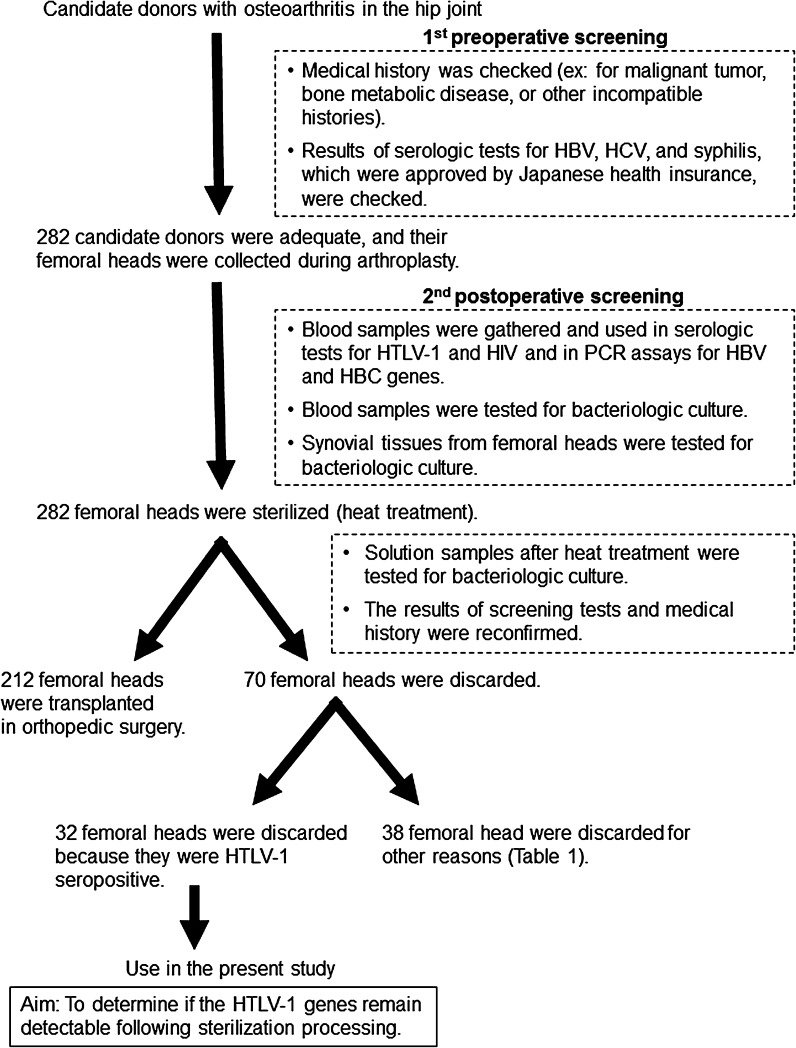
Study design and screening process. We carried out two steps of screening tests for candidate allograft donors. Among the 282 candidate surgical donors, 32 donors (11.3 %) were positive for HTLV-1 antibody. We used the femoral heads from seropositive candidates in the present study. HBV, hepatitis B virus; HCV, hepatitis C virus; HTLV-1, human T-lymphotropic virus type 1; HIV, human immunodeficiency virus

Between 2008 and 2012, we collected 282 femoral heads at Kagoshima’s regional bone bank from living donors with osteoarthritis of the hip joint. In candidate donors who planned to undergo total hip arthroplasty because of osteoarthritis, preoperative screening was performed according to each donor’s medical history and serologic test results (screening for hepatitis B virus, HCV, and syphilis). Furthermore, we performed postoperative screening of the donors by performing serologic tests for human immunodeficiency virus and HTLV-1 as well as PCR tests to detect hepatitis B virus and HCV genes (Fig. 1). We carried out blood cultures and cultures of the synovium tissue to detect bacterial infection, and we excluded all femoral heads with positive samples. The procured femoral heads were disinfected by heat treatment using the Lobator SD-2 Bonebank System (Telos GmbH, Marburg, Germany) (Hofmann et al. 1996). The heat treatment condition was 82.5 °C as measured within the bone, plateau phase 15 min, closed system without additional pressure, and treatment totally for 94 min.

After the heat treatment, the resulting solutions were cultured to confirm the efficacy of the thermal disinfection. We finally reconfirmed the results of the screening tests and medical history; based on these findings, we discarded 70 femoral heads.

The femoral heads from 32 donors were not used for transplantation because the donors were found to be seropositive for HTLV-1. In order to verify the existence of HTLV-1 provirus in the femoral heads, DNA was extracted from the bone marrow tissue from these femoral necks, which included many blood cells, by using a DNeasy tissue kit according to the manufacturer’s instructions (QIAGEN, Tokyo, Japan). The bone marrow tissue, including the bone itself, was crushed into pieces before the DNA extractions were performed (Thomas and Moore 1997).

100 ng of the extracted DNA was subjected to real-time PCR with an ABI 7700 sequence detection system (Applied Biosystems, Tokyo, Japan). HTLV-1 tax primers and TaqMan probes were used for the real-time PCR using β-actin as an internal control. (Matsuura et al. 2008). The assay was performed in triplicate, and the copy numbers were determined by standard curves. The proviral load per 10^4^ cells was calculated as follows:

(mean of tax copy number)/(mean of β-actin copy number) × 2 × 10^4^.

## Results

Among the 282 procured femoral heads, 212 were used for allografts in orthopedic surgery. However, 70 femoral heads (24.8 %) were discarded for various reasons (Fig. 1). The main reason for discarding a femoral head was serologic HTLV-1-positivity (45.7 %) in the discarded femoral heads (Table 1).

**Table 1:** The frequency of each reason for discarding femoral heads

Discard reason	Rate
HTLV-1Ab positive	45.7 %
Omission of bacteriologic culture	21.4 %
Blood culture bacterial contaminations	7.1 %
Malignant tumor (became clear later)	5.7 %
Poor bone quality	5.7 %
Inadequate documentation	5.7 %
Others infection	4.3 %
Others	4.3 %

Among the 282 candidate surgical donors, 32 donors (11.3 %) were positive for HTLV-1-specific antibody. All donors less than 50 years old were anti-HTLV-1 antibody-negative, whereas 14.2 % of the donors who were at least 50 years old were HTLV-1-positive (Table 2). Among the 32 femoral heads from anti-HTLV-1 antibody-positive candidate donors, DNA was sufficiently extracted from three femoral heads. An efficient DNA extraction was not possible in the other 29 femoral heads. Notably, we were able to detect HTLV-1 genes by PCR in all three of the DNA extraction samples from post-processing femoral heads.

**Table 2:** Human T-cell leukemia virus type 1 (HTLV-1)-specific antibody-positive rates in surgical donors, by age

Age (years)	HTLV-1 Ab (−)	HTLV-1 Ab (+)	Total	Rate (%)
30–39	1	0	1	0.0
40–49	24	0	24	0.0
50–59	61	9	70	12.9
60–69	49	3	52	5.8
70–79	87	14	101	13.9
80–89	28	6	34	17.6
Total	250	32	282	11.3

*Ab* antibody

## Discussion

Japan is an endemic area for HTLV-1. According to blood donor screening data in Japan for 2006 and 2007, the HTLV-1 prevalence rates are 0.66 and 1.02 % in men and women, respectively, and 1 million individuals are carriers of HTLV-1 (Satake et al. 2012). Japan has an uneven distribution of HTLV-1 prevalence, and 45.7 % of all carriers live in the Kyushu region, which is the southern tip of the Japanese archipelago. Overall, the seropositive rate of HTLV-1 is highest in the Kagoshima Prefecture (1.95 %). In 1997, Kagoshima governments organized the Kagoshima ATL Prevention Committee, and infection control for mother-to-infant transmission was conducted. Accordingly, the HTLV-1 seropositive rate in blood donors tended to decrease to <1.0 % (Uchiyama 2006).

In spite of the lower HTLV-1 seropositive rate in blood donors, the anti-HTLV-1 antibody-positive rate in the donors to our bone bank was 11.3 %, which is higher than that in blood donors, particularly among those over 50 years old. Allograft candidate donors with osteoarthritis are largely elderly, and, thus, they were not included in the mother-to-infant transmission infection control program. This may be one reason for the high prevalence of HTLV-1 among bone bank donor candidates.

HTLV-1 is a retrovirus that enters the nucleus of human T cells and exists as integrated proviral DNA in host genomic DNA (Yoshida et al. 1984). Cell-to-cell contact is necessary to facilitate viral infection, and infection via a free virus particle is extremely inefficient (Fan et al. 1992). Therefore, HTLV-1 is commonly transmitted through blood transfusions, which may include infected lymphocytes, but it is seldom transmitted through blood plasma components and blood preparations. However, Sanzén and Carlsson reported a case of HTLV-1 transmission through transplantation of a fresh-frozen unprocessed femoral head (Sanzén and Carlsson 1997).

Our results show that viral genes exist in the preserved bones derived from HTLV-1 carriers, even though these bones had been processed using a commercial thermal bone banking device. Among 32 femoral heads, we were only able to extract DNA from three of them. The fact that HTLV proviral DNA was amplified in 3 out of 32 samples only may indicate that the inactivation procedure considerably degrades cellular DNA (including integrated proviral DNA). Alternatively, the PCR demonstration of proviral DNA may just indicate DNA from dead cells. Our detection of HTLV-1 genes in all three post-processing samples demonstrates that viral genes can remain in bone marrow lymphocytes in spite of processing. The heat treatment reduced the risk of virus transmission by inactivating the infected cells (Nowak et al. 1993; Pruss et al. 2001). Any transmission of HTLV is highly unlikely since both, virus and T cells, are highly heat sensitive. However, we cannot deny possible viral infection if infected lymphocytes keep the viability after inadequate processing. Therefore, the heat treatment needs the condition that be able to disinfect the virus completely.

These findings emphasize the importance of screening donors for HTLV-1 prior to the use of their donations in transplantation (Schreiber et al. 1996; von Garrel et al. 1997). The limitation of this study was that we didn’t confirm the presence of viable cells including proviral DNA in our samples.

HTLV-1 screening in endemic areas such as Kagoshima is especially important. Because HTLV-1 seroprevalence is high in Kagoshima, we have performed serologic tests for HTLV-1 in candidate donors as part of their preoperative screening since 2013. Preoperative screening for HTLV1 eliminates the need for later screening tests, improving the cost-effectiveness of bone allografts.

We detected HTLV-1 genes in samples from bone tissue that had been through sterilization processing. Therefor, it is important that the virus was completely disinfected by heat treatment. Additionally, our data reconfirm the importance of HTLV-1 screening at bone banks, particularly in endemic areas such as southwest Japan.
